# Development of Pelubiprofen Tromethamine with Improved Gastrointestinal Safety and Absorption

**DOI:** 10.3390/pharmaceutics13050745

**Published:** 2021-05-18

**Authors:** Ji Yeon Park, Dong Ho Oh, Sang-Wook Park, Bo Ram Chae, Chul Woo Kim, Sang Heon Han, Hyeon Jong Shin, Soo Bin Yeom, Da Yeong Lee, Min Kyu Park, Sang-Eun Park, Jun-Bom Park, Kyung-Tae Lee

**Affiliations:** 1 Department of Pharmaceutical Biochemistry, College of Pharmacy, Kyung Hee University, 26 Kyungheedae-ro, Dongdaemun-gu, Seoul 02447, Korea; jyp100@daewonpharm.com (J.Y.P.); qkrtkddms0930@naver.com (S.-E.P.); 2 Daewon Pharm. Co., Ltd., 520 Cheonhodae-ro, Gwangjin-gu, Seoul 04994, Korea; frank82@daewonpharm.com (D.H.O.); swpark@daewonpharm.com (S.-W.P.); hifyram@daewonpharm.com (B.R.C.); kimcw87@daewonpharm.com (C.W.K.); shhan@daewonpharm.com (S.H.H.); shoot24@daewonpharm.com (H.J.S.); ysubin0111@daewonpharm.com (S.B.Y.); dayoung717@daewonpharm.com (D.Y.L.); 3 Department of Clinical Pharmacology and Therapeutics, Chungbuk National University, 1 Chungdae-ro, Seowon-gu, Cheongju, Chungbuk 28644, Korea; mkparkdau@gmail.com; 4 Department of Life and Nanopharmaceutical Science, College of Pharmacy, Kyung Hee University, 26 Kyungheedae-ro, Dongdaemun-gu, Seoul 02447, Korea; 5 College of Pharmacy, Sahmyook University, 815 Hwarang-ro, Nowon-gu, Seoul 01795, Korea

**Keywords:** pelubiprofen tromethamine, solubility, permeability, gastrointestinal safety, absorption

## Abstract

Pelubiprofen (PEL), which is a commercialized non-steroidal anti-inflammatory drug (NSAID), is associated with the risk of gastrointestinal (GI) adverse events following long-term exposure and has poor water-soluble properties. Here, a new pelubiprofen tromethamine (PEL-T) with improved solubility, permeability, GI safety, and absorption, compared to PEL, has been developed. The nuclear magnetic resonance spectroscopy (NMR), differential scanning calorimetry (DSC), and Fourier transform infrared spectroscopy (FT-IR) results confirmed that the PEL-T was well formed. The powder of PEL-T showed the presence of additional 6H protons at δ 3.66–3.61 in the ^1^H NMR spectrum, and shifted the sharp endothermic peaks at 129 °C in DSC, and the spectrum of distinct absorption peaks in FT-IR. In addition, compared with PEL, PEL-T showed a significantly improved solubility in various media and an increased permeability coefficient (K_p_) in Caco-2 cells. Furthermore, compared to PEL oral administration, PEL-T was found to significantly reduce the damaged area in an acute gastric damage rat model. The pharmacokinetic study of the PEL-T powder showed higher maximum plasma concentration (C_max_) and area under the plasma concentration–time curve from 0 h to the last time point (AUC_t_) than those of the PEL powder. Taken together, our data suggest that PEL-T is a recommendable candidate with enhanced gastrointestinal safety and better absorption compared with commercial PEL.

## 1. Introduction

Pelubiprofen (2-[4-(2-oxocyclohexylidene methyl) phenyl] propionic acid, PEL) is one of the 2-arylpropionic acid class members of non-steroidal anti-inflammatory drugs (NSAIDs). It has been used for the treatment of osteoarthritis, rheumatoid arthritis (RA), backache, and for its antipyretic effect in acute upper respiratory infections [1]. Its pharmacological effect is mediated by the dual inhibition of selective cyclooxygenases (COX)-2 activity (IC_50_ ratio of COX-1 and COX-2 is 3.7:1) and lipopolysaccharides (LPS)-induced inflammatory gene expression via nuclear factor kappa-light-chain-enhancer of activated B cell (NF-κB) inactivation [2]. PEL is a poorly soluble and biopharmaceutical classification system (BCS) II drug and is absorbed in the gastrointestinal (GI) tract after oral administration, reaching the maximum plasma concentration (C_max_) after 30 min in rats, and after 1 and 2 h in male and female beagle dogs, respectively [3,4]. PEL exerts its pharmacological activity by forming an active metabolite in the body after being absorbed in the small intestine [5]. The relatively selective effects on COX-2 activity and its prodrug property contribute to its less GI ulcerogenicity than other non-selective NSAIDs.

However, the long-term exposure of NSAIDs frequently leads to GI adverse events, such as dyspepsia, abdominal pain, gastric ulcers, and bleeding [6,7]. Therefore, a COX-2 selective inhibitor was developed to improve this unsatisfactory profile of NSAIDs and it is commonly used in the elderly and in those who are at risk of GI bleeding, including patients with RA [8,9,10]. It should be noted that these strategies are not GI-safe enough in high-risk patients. In clinical studies and based on post-marketing surveillance experience, PEL was found to cause GI adverse events; despite that, the COX-2 selective inhibitors have better GI safety compared to conventional NSAIDs [1,11,12,13]. The prescription information of Pelubi^®^ (pelubiprofen) and Celebrex^®^ (celecoxib) mentions GI-adverse events, such as heartburn, dyspepsia, abdominal pain, and GI bleeding, and is not recommended for patients with peptic ulcer or GI bleeding [1,11]. Therefore, for the long-term use of PEL, research on interventions that can reduce the GI adverse events is needed.

Salt formation provides a means of changing the physicochemical and resulting biological properties of a drug molecule without changing the structure of the pharmacologically active moiety. However, the selection of an appropriate salt form that has desirable properties, such as solubility, stability, permeability, and bioavailability, plays an important role in the design and development of the drug [14]. PEL is a relatively weak acid drug with a pKa of 5.0–5.3 that is practically insoluble in water and has low solubility in acidic media. The low water solubility of the active pharmaceutical ingredient (API) is the major drawback of the formulation because it leads to a very low dissolution rate and consequently, to a low GI absorption [15]. Meanwhile, the bioavailability of BCS II drugs can be sufficiently increased through solubilization; in other words, the solubility may be a limiting factor in the development of alternative dosage forms or delivery systems [16]. Salt formation is a method that can be applied to increase the aqueous solubility and dissolution rate, which facilitates the rapid absorption of the salt across the gastric and enteric mucosa [17,18,19].

To improve the solubility and prevent the risk of GI-adverse events of PEL, pelubiprofen tromethamine (PEL-T) was selected by manufacturing processes involving cooling, anti-solvent addition, or evaporation using various metals, amines, and amino acid candidates (data not shown).

In this study, the powder characteristics of PEL-T were investigated via structural analysis, solubility, and permeability analyses, and a study to improve its GI safety was conducted in rats. Finally, the pharmacokinetic (PK) profiles of PEL-T powder in rats were evaluated and compared with those of PEL powder, which was used as a reference.

## 2. Materials and Methods

### 2.1. Materials

PEL and tromethamine (tris(hydroxymethyl)aminomethane, THAM) were purchased from SAMOH Pharm. Co., Ltd. (Siheung, Korea) and Merck KGaA (Darmstadt, Germany), respectively. Methanol and acetone were purchased from Avantor, Inc. (J.T. Baker^®^, Phillipsburg, NJ, USA). All other chemicals and reagents used were of reagent grade and were used without further purification. Water was purified using a Milli-Q purification system (Millipore Corp., Bedford, MA, USA).

### 2.2. Preparation of the PEL-T Powder

First, 5 g of PEL were dissolved in 25 mL of methanol, and 2.35 g of THAM were added and stirred at 40 °C for 1 h. After cooling to a controlled room temperature (CRT; 20–25 °C), methanol was concentrated under reduced pressure (350 mmHg) conditions. A volume of 70 mL of acetone was slowly added to the concentrate, stirred at CRT for 2 h, and filtered through a 0.45 μm membrane filter. The filtrate was washed with 100 mL of a mixture of methanol and acetone at a ratio of 1:3 (*v*/*v*), and the resultant solid was vacuum-dried at 40 °C for 12 h.

### 2.3. Physico-Chemical Properties of PEL and PEL-T Powder

^1^H NMR spectra were recorded in methanol-*d_4_* on a spectrometer (Bruker Avance II 500 MHz spectrometer, Karlsruhe, Germany) using tetramethylsilane as an internal standard (IS), and the chemical shifts are given in δ (ppm). Additionally, the ^1^H NMR and ^13^C NMR spectra were recorded in dimethyl sulfoxide (DMSO)-*d_6_* on a spectrometer (Bruker Avance III HD 400 MHz spectrometer, Karlsruhe, Germany) (Tables S1 and S2, Figures S1 and S2). The thermal transition properties were investigated using a differential scanning calorimeter (TA DSC Q20, New Castle, DE, USA). Each sample was transferred to a sealed aluminum pan and heated at a rate of 10 °C/min from 40 °C to 250 °C under nitrogen purging. The chemical structures were also evaluated using Fourier transform infrared spectroscopy (Nicolet iS10 FT-IR Spectrometer, Madison, WI, USA). Each spectrum was obtained over the wavelength range of 4000–450 cm^−1^ with 32 scans at a resolution of 4 cm^−1^.

### 2.4. HPLC Determination of PEL

PEL and butylparaben (IS) were prepared and further diluted in a mixture of methanol and deionized water at a ratio of 4:1 (*v*/*v*) to provide a working PEL and IS solution of concentration 30 µg/mL. The PEL concentration was determined using an HPLC system (Model 1260; Agilent Technologies Inc., Santa Clara, CA, USA). Chromatographic separation was performed using a C_18_ column (4.6 × 150 mm, 5 µm, Waters, Milford, MA, USA) at a flow rate of 1.1 mL/min with the mobile phase consisting of methanol, water, and acetic acid at a ratio of 1200:800:1 (*v*/*v*). The sample was injected into the column, and the peaks were monitored using a UV detector at 274 nm.

### 2.5. Solubility, Dissolution, and Stress Stability Test

The solubilities of PEL-T and PEL were compared and evaluated based on the pH changes. After dissolving 5 g of PEL-T and PEL each in 5 mL of water, a pH 1.2 solution, a pH 4.0 solution, and a pH 6.8 solution, respectively, the mixtures were stirred for 24 h, filtered using a 0.45 μm polyvinylidene difluoride (PVDF) syringe filter, and then were analyzed using an HPLC assay, as described above (Section 2.4).

The release of PEL-T and PEL powders was studied using a USP dissolution apparatus II (Agilent 708-DS; Agilent Technologies Inc., Santa Clara, CA, USA) and an automatic sampling system. The dissolution media (900 mL) tested were formulated to pH 1.2 (2.0 g of NaCl, 7.0 mL of HCl, and water to 1000 mL), pH 4.0 (0.05 mol/L acetic acid-sodium acetate buffer solution at a 41:9 ratio), pH 6.8 (6.8 g of KH_2_PO_4_, 0.94 g of NaOH, and water to 1000 mL), and water maintained at 37 ± 0.5 °C. The paddle speed was set at 50 rpm. After each sample containing 45 mg of PEL equivalent was put in the vessel, aliquots (5 mL) were withdrawn at predetermined time points (0.083, 0.167, 0.25, 0.5, 0.75, 1, 1.5, 2 h), and replenished with fresh medium (5 mL). The samples were analyzed using the HPLC assay described above (Section 2.4).

Stability tests of PEL-T and PEL were performed under stress storage conditions. PEL-T and PEL were packed into high-density polyethylene (HDPE) bottles with caps and used as stability test samples. For stress stability, the samples were stored at a high temperature (80 ± 5 °C) or high humidity (relative humidity (RH) 90 ± 5%). The drug content and impurities were determined using the HPLC assay described above (Section 2.4) at the following time points: 0, 14, and 21 days (Tables S3 and S4).

### 2.6. Permeability Test in Caco-2 Cells

Caco-2 monolayer experiments were performed as previously described with a few modifications [20]. The Caco-2 cells were obtained from the Korean Cell Line Bank (Seoul, Korea) and used in the transport experiments between passages 40 and 50. The cells were cultured at 37 °C with 5% CO_2_ and maintained in Dulbecco’s modified Eagle’s medium with 10% fetal bovine serum, 1% nonessential amino acids, 10,000 units/mL penicillin, and 10,000 μg/mL streptomycin. For the transport studies, cells were seeded on rat tail collagen-coated Transwell insert filters on 12-well plates at 3 × 10^5^ cells/500 μL and were cultured for 21–29 days. For the first 7 days, the medium was replaced every 2–3 days, and daily thereafter. Monolayer integrity was checked before and after each experiment using a Millicell-ERS instrument to determine the transepithelial electrical resistance (TEER) across the monolayer and to ensure that there were no substantial changes in the monolayer integrity throughout the duration of the experiments.

Prior to transport experiments, the cell monolayers were washed twice using Hank’s balanced salt solution (HBSS), and the Caco-2 cell Transwell model was preincubated at 37 °C for 15 min. For the measurement of absorptive (for apical to basolateral [A to B] transport) drug transport, 0.4 mL of transport media containing HBSS (with PEL and PEL-T at a final concentration of 50 μM as PEL, *n =* 5) was added to the apical side of the cell monolayer and 1.2 mL of transport media containing HBSS were added to the basolateral side. The inserts were transferred to a new well in which 1.2 mL of transport media were added in advance after 15, 30, 60, and 120 min, and the permeate sample was collected from the basolateral side. The sample was pretreated using a protein precipitation method and the concentrations of PEL in each sample were determined using an LC–MS/MS assay, as described below (Section 2.9). For the measurement of secretory (for basolateral to apical [B to A] transport) drug transport, 1.2 mL of transport medium containing HBSS (with PEL and PEL-T of a final concentration of 50 μM as PEL, *n =* 5) were added to the basolateral side, and 0.4 mL of transport media containing HBSS was added to the apical side. A 0.2 mL aliquot of transport media was collected from the apical side after 15, 30, 60, and 120 min. After the removal of each sample, the volume removed (0.2 mL) was replaced with fresh transport media containing HBSS. The sample was pretreated using a protein precipitation method and the concentration of PEL in each sample was determined using an LC–MS/MS assay. The steady state flux (J_s_) and permeability coefficient (K_p_) were calculated according to Equations (1) and (2):

J_s_ [ng/(cm^2^ × h)] = Q_r_/(A × t) (1)

K_p_ (cm/h) = J_s_/C_d_ (2)

where Q_r_ is the total permeation amount of PEL in the basolateral (for the absorptive transport) or apical (for the secretory transport) side (ng), A is the surface area of the insert (1.12 cm^2^), t is the permeation time (h), and C_d_ is the initial drug concentration in the apical (for the absorptive transport) or basolateral (for the secretory transport) side (ng/cm^3^). The efflux ratio (ER) was calculated according to Equation (3):

ER = K_p_ [B to A]/K_p_ [A to B] (3)

### 2.7. Gastric Mucosal Injury Test in Rats

The gastric mucosal injury test was performed by KNOTUS Co., Ltd. (Guri, Korea) and approved by the Animal Ethics Committee (Protocol number: KNOTUS IACUC 18-KE-354 approved 18 October 2018). Male Sprague-Dawley (SD) rats (7-weeks-old) from Orient bio Co., Ltd. (Gapyeong, Korea) were maintained under constant laboratory conditions (temperature: 23 ± 3 °C, RH: 50 ± 15%, light/dark cycle: 12 h). The rats were randomly divided into three groups, each group consisting of 10 animals. PEL and PEL-T were dissolved in a 0.5% carboxymethylcellulose sodium (CMC) solution in distilled water before use. PEL (300 mg/kg), PEL-T (440.7 mg/kg), or vehicle (0.5% CMC) was administered orally to rats (*n* = 10/group) after 48 h of fasting. After 6 h, the rats were sacrificed via cervical dislocation, their stomachs were removed, and the gastric mucosa was photographed using a digital camera. The area of the lesions was analyzed using ImageJ software (National Institutes of Health, Bethesda, MD, USA).

### 2.8. PK Studies in Rats

The PK tests in rats were performed at the Croen Inc. (Suwon, Korea) and approved by the Animal Ethics Committee (Protocol number: C20RC-124N/20R051 approved 16 June 2020). Male Sprague-Dawley (SD) rats (8-weeks-old) from DBL Co., Ltd. (Eumseong, Korea) were maintained under constant laboratory conditions (temperature: 22 ± 3 °C, RH: 50 ± 20%, light/dark cycle: 12 h). The rats were divided into two groups, each composed of 10 rats. The animals were fasted overnight and were only allowed free access to water. PEL powder and PEL-T powder in aqueous solutions were administered orally to the rats at an equivalent PEL dose of 9.3 mg/kg (90 mg daily dosage converted to volume ratio). Blood samples (500 μL) were collected directly from the jugular vein at predetermined time points (0, 0.083, 0.167, 0.25, 0.5, 0.75, 1, 2, 3, 4, and 8 h) using heparinized syringes. The collected blood samples were centrifuged immediately at 12,000 rpm for 5 min at 4 °C and the obtained plasma samples were stored at −70 °C until analysis.

### 2.9. LC-MS/MS Method and PK Studies

The plasma concentration of PEL was determined using LC–MS/MS (Agilent 1260; Agilent Technologies Inc., Santa Clara, CA, USA). The chromatographic separation of PEL was performed using an ODS column (Poroshell 120 EC-C18, 2.1 × 30 mm, 2.7 µm, Agilent Technologies Inc., Santa Clara, CA, USA). The mobile phase (MP) consisted of 0.1% formic acid and acetonitrile with a gradient elution and the flow rate used was 0.4 mL/min. Mass spectrometric detection was done using an AB Sciex API 4000 triple quadrupole mass spectrometer (AB Sciex Instruments, Framingham, MA, USA). The electrospray ionization (ESI) source was set to positive ion mode. The mass-to-charge ratios (*m*/*z*) used were 259.0 → 195.2 for PEL and 255.1 → 208.9 for ketoprofen, which were used as the internal standards (IS). The calibration curves were prepared at concentration values of 0.5–750 ng/mL and showed linearity with a coefficient of determination (r^2^) value greater than 0.99. The assay method was validated for linearity, accuracy, and precision.

The PK parameters for PEL were calculated using the noncompartmental analysis and WinNonlin^®^ 8.1 (Pharsight, CA, USA). The area under the plasma concentration–time curve from 0 h to the last time point (AUC_t_) was calculated for all samples using the linear trapezoidal method. The maximum plasma concentration and time to reach the maximum plasma concentration (T_max_) were directly obtained from the plasma concentration–time profiles.

### 2.10. Statistical Analysis

All data are expressed as mean ± standard deviation (S.D.). Statistical analysis was performed using Student’s *t*-test or one-way ANOVA and Tukey’s multiple comparisons test using Prism 7.04 (GraphPad Software Inc., San Diego, CA, USA). The differences were considered significant at a *p*-value *<* 0.05, unless otherwise indicated.

## 3. Results and Discussion

### 3.1. Physicochemical Characteristics of PEL-T

Table 1 and Figure S1 show the ^1^H NMR spectra of PEL and PEL-T. The carboxylic acid proton (h) of the PEL and the hydroxyl (i) and ammonium (j) protons of the PEL-T were not observed in methanol-*d_4_* solvent due to deuterium exchange. However, the ^1^H NMR spectra of PEL-T tested under different conditions (in DMSO-*d_6_* at 400 MHz; Figure S2a) showed the presence of exchangeable protons. The hydroxyl groups and the amine group (i) protons of the PEL-T can be observed in DMSO-*d_6_* solvent at δ 5.33 (5H, br s). The overall patterns of the ^1^H NMR spectra of PEL and PEL-T were similar, as shown in Table 1. The big difference was the presence of additional 6H protons at δ 3.66–3.61 in the ^1^H NMR spectrum of PEL-T, indicating the formation of THAM salt. In addition, two types of carbon signals at δ 61.00 (3C) and δ 60.21 (1C) in the ^13^C-NMR spectra of PEL-T (in DMSO-*d_6_* at 400 MHz; Figure S2b) corresponding to THAM are shown, which is indicative of salt formation.

**Figure 1:**
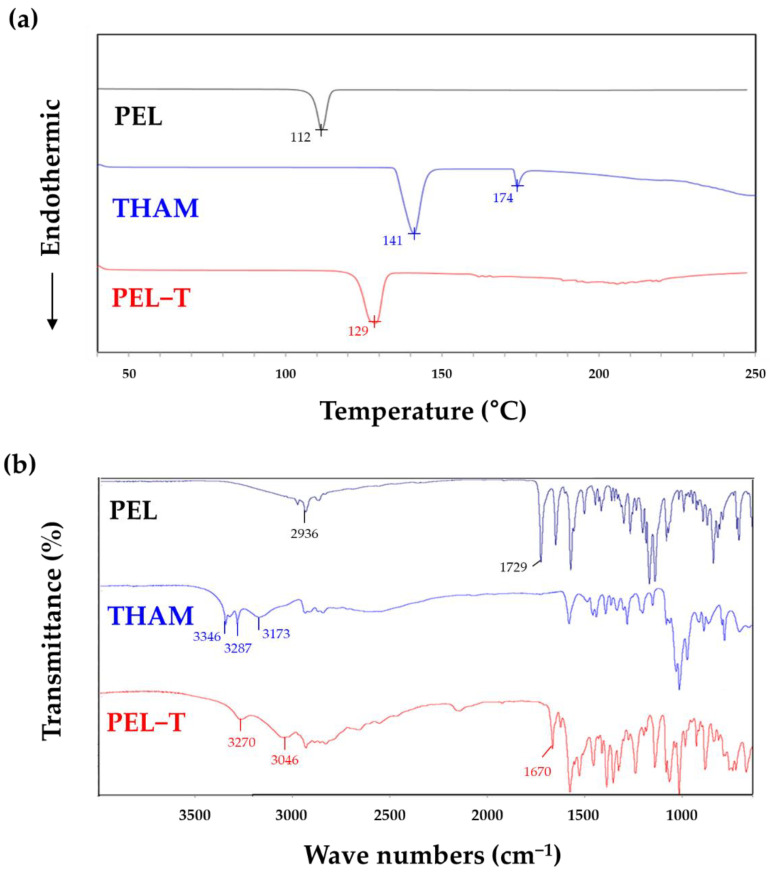
Structural characteristics of PEL-T. (**a**) DSC thermograms; (**b**) Fourier-transform infrared (FT-IR) spectra. PEL (black), THAM (blue), and PEL-T (red).

According to the DSC thermograms in Figure 1a, the melting point of PEL-T at 129 °C is distinctive and very different from the melting point of PEL at 112 °C and the two endothermic peaks of THAM at 141 °C and 174 °C [17]. It was confirmed that PEL-T is not a simple mixture of PEL and THAM, but a new crystalline salt. The formation of THAM salt in the PEL-T was confirmed by the FT-IR spectra, as shown in Figure 1b. The O–H bond in the PEL-T was confirmed at 3270 cm^−1^, which was not observed for PEL. This intense O–H bond signal derives from the THAM absorption peak shift at 3346 and 3287 cm^−1^ [21,22]. In addition, the disappearance of the sharp peak of the O-H stretch at 2936 cm^−1^ in PEL and a strong asymmetric −COO^−^ stretching vibration at 1670 cm^−1^ for the carboxyl salts in PEL-T were identified, showing a shift of the PEL absorption peak at 1729 cm^−1^ for the C=O stretch of a carboxylic acid group [23]. Furthermore, a number of strong bands presented in the range of 3046–2000 cm^−1^ in the spectrum of PEL-T are expected to be generated from overtones of protonated amine groups and the combination of stretching vibrations, whereas the N-H stretching vibration in the THAM absorption peak was observed at 3400–3100 cm^−1^ [17,22]. Furthermore, the FT-IR spectrum of PEL-T showed additional IR bands at 1000 cm^−1^, which corresponds to the C-O stretching vibrations, again confirming the presence of alcohol functional group of THAM moiety in the molecule.

### 3.2. Solubility and Dissolution Behavior of PEL-T

**Figure 2:**
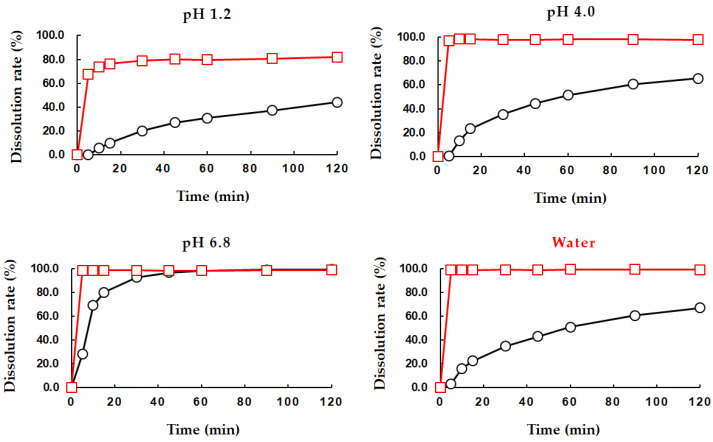
Dissolution profiles of PEL powder (black) and PEL-T powder (red) in the paddle apparatus at 50 rpm in 900 mL of buffer media at pH 1.2, 4.0, 6.8, and water (*n* = 6).

As shown in Table 2 and Figure 2, the solubility of PEL-T was much improved, and the dissolution rate was higher than that of PEL in all conditions. PEL-T showed a 20,000-times higher solubility than PEL in pH 1.2 media, and about 100 times higher solubility than PEL at pH 6.8. PEL is a relatively weak acid drug with pKa of 4.6, which means that the solubility of the drug increases as the pH increases. However, the formation of THAM salt of PEL resulted in an increase in solubility, regardless of the pH of the medium. The dissolution results (Figure 2) showed that there was a very large difference in the dissolution rate of PEL according to the pH, while PEL-T showed a relatively high dissolution rate. The excellent solubility and high dissolution rate of the THAM salt of PEL in various pH conditions suggest the possible development of simple and diverse pharmaceutical formulations. In the case of poorly soluble drugs, pharmaceutical strategies such as solubilization technology or grinding technology to fine particles need to be used, in this order, to increase the bioavailability; however, it is expected that PEL-T will be able to reach the maximum bioavailability using very simple and common pharmaceutical manufacturing processes. As a result, it is possible to reduce the tablet size and weight with minimized usage of excipients in order to lower the manufacturing cost. For example, the application of THAM salt to Ketorolac or Fosfomycin for improving solubility has been reported [24,25]. This salt is highly water soluble and can be administered through various routes, such as intravenous, subcutaneous, oral, and intramuscular administration. This is the only NSAID currently available as a nasal spray.

Moreover, a stress stability test of PEL-T and PEL was performed at high temperature (80 ± 5 °C) or high humidity (RH 90 ± 5%) and the changes in the drug contents and degradation products were analyzed (Tables S3 and S4). No significant changes in the drug contents of PEL-T and PEL were observed throughout the entire period of the stress stability test, despite the increased solubility of PEL-T.

### 3.3. Permeability Test of PEL-T in Caco-2 Cells

As shown in Table 3, we evaluated the bi-directional permeability coefficients (K_p_) as PEL crossed the Caco-2 cell monolayers. As a result of measuring the TEER values before and after the permeability test in Caco-2 cells, the recovery value was higher than 96% compared to the initial value in both A to B and B to A directions, and no cell morphology problems were found, even when cells were observed under a microscope. The K_p_ of PEL-T in the absorptive [A to B] direction was significantly higher (1.2 times higher) than that of PEL. The K_p_ of PEL in the secretory [B to A] direction was 15.57, whereas that of PEL-T was 11.65, being 1.3 times higher in PEL. This, however, was not statistically significant. It can be observed that the absorption of PEL-T across intestinal cells was increased. In addition, the high secretion of PEL from B to A was shown and the efflux ratio values of PEL and PEL-T were 1.24 and 0.76, respectively. It was found that the efflux ratio of PEL was lower than 2, which indicated an active efflux [26], but was 1.6 times higher than that of PEL-T. These results suggest that PEL-T may increase absorption and bioavailability in vivo.

### 3.4. PEL-T Reduced Gastric Mucosal Injury in Rats

**Figure 3:**
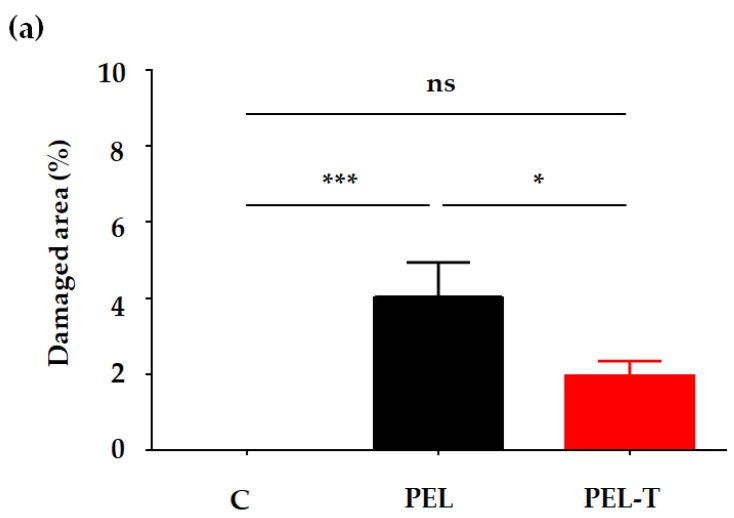

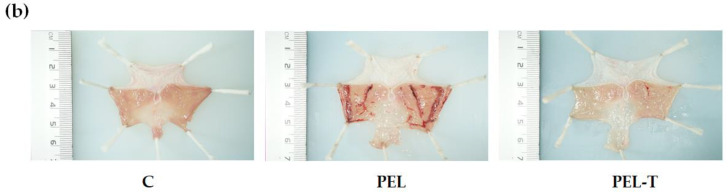
(**a**) Damage area (%) in the gastric mucosal injury test of rats, which was assessed using the image program (ImageJ software). Each value represents the mean ± S.D. (*n* = 10) (*** *p <* 0.001 level compared to C; * *p <* 0.05 level compared to PEL; ns, no significant difference). (**b**) Representative gastric mucosal lesion photographs of each group; C, normal control.

To investigate whether the gastric mucosal damage of PEL-T is comparable to that of PEL, we established an acute gastric damage model of PEL and PEL-T in male SD rats. As shown in Figure 3a, the administration of PEL (300 mg/kg, p.o.) showed significant (*p <* 0.001) induction of mucosal hemorrhagic lesions (4.01%), compared with the vehicle-treated control group. However, the administration of PEL-T (440.7 mg/kg, p.o., equivalent PEL dose of 300 mg) showed a reduced mucosal damaged area (1.95%) with an inhibition of 51.4%, compared with administration of PEL, and this difference was statistically significant (*p <* 0.05). Photographs of the lesions confirmed that the administration of PEL showed more mucosal hemorrhagic lesions (Figure 3b). Oral administration of PEL-T reduced the gastric damage compared with that resulting from PEL administration. As THAM has been used as a treatment for metabolic acidosis along with sodium bicarbonate [27], it inhibits rapid changes in gastric pH and neutralizes stomach acidity. Therefore, the mucosal damage caused by PEL-T was observed to be less potent than that caused by PEL at the same dose of 300 mg/kg. THAM salt was found to apparently decrease gastric irritation by improving the water solubility, which reduces the local concentration of the drug in the stomach, as has been reported in the case of dexketoprofen [28].

### 3.5. Comparative PK in Rats

**Figure 4:**
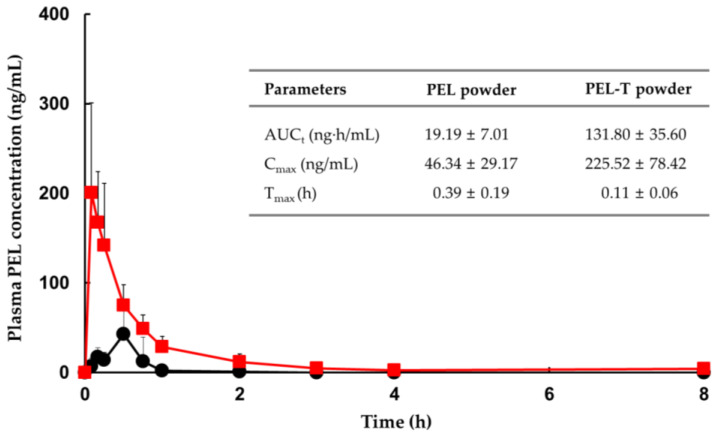
Mean (±S.D.) plasma concentration–time profiles of PEL in the plasma samples after a single oral administration of PEL-T (red) or PEL (black) powder to rats. PK parameters are summarized in the inset table. The data points and error bars represent the mean ± S.D. (*n* = 10).

Figure 4 shows the mean ± S.D. plasma concentration–time profiles and PK parameters of the oral administration of PEL and PEL-T powder to rats. Noncompartmental methods were used to calculate the following PK parameters of PEL: AUC_t_, C_max_, and T_max_. PEL-T was rapidly converted to PEL in the blood and reached the maximum plasma concentration in less than 10 min. The C_max_ and AUC_t_ of PEL-T powder were 4.9 and 6.9 times higher than those of PEL powder, respectively. Based on these results, this higher absorption of PEL-T was caused by the increased solubility and permeability. Similarly, many reports have demonstrated that the absorption enhancement can generally be correlated with the solubility and/or permeability of a drug [29,30,31]. According to the BCS evaluation guidelines by the Food and Drug Administration (FDA), the solubility, rapid dissolution ability, and biological permeability of a drug are the main factors that affect its bioavailability [32]. Three pharmaceutical salts/cocrystals of enoxacin (EX) with oxalic acid, malonic acid, and fumaric acid could enhance the antibacterial activity of the corresponding parent compound (EX) by improving its solubility and permeability [30]. As fenofibrate is highly lipophilic, virtually insoluble in water, and poorly absorbed, coadministration with meals is necessary to maximize the bioavailability of older formulations [31]. To overcome these drawbacks, micronized and nanoparticle formulations of fenofibrate with reduced particle sizes were developed, resulting in greater solubility and improved bioavailability [33,34]. A recently introduced hydrophilic choline salt of fenofibric acid can be taken without coadministration with meals and has the highest bioavailability among the marketed formulations [35]. By adding choline salt to fenofibrate, the dose of the active substance was lowered from 200 mg to 135 mg.

## 4. Conclusions

We successfully developed PEL-T as a salt of PEL with improved solubility, permeability, and GI safety, compared to PEL. Consequently, PEL-T powder gave significantly higher AUC_t_ and C_max_ values than PEL in rats. Therefore, as an alternative salt, PEL-T is a recommendable candidate with improved GI safety and absorption, compared to the commercial PEL. For the development of a dosage form of PEL-T, detailed studies of the pharmaceutical substance by pharmacopoeial requirements and pharmacokinetic studies in volunteers are required.

## 5. Patents

Korea Patent KR-10-2019-0055671.

## Figures and Tables

**Table 1:** ^1^H NMR (500 MHz, methanol-*d4*) chemical shifts (δ), multiplicities, and assignments of PEL and PEL-T.

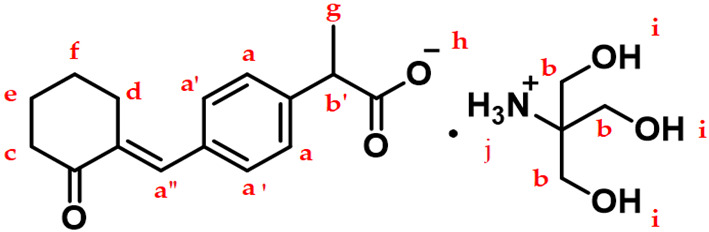		
Assignments	PEL	PEL-T
a, a’, a”	7.44–7.36 (m, 5H)	7.46–7.38 (m, 5H)
b’, b	3.78–3.72 (m, 1H)	3.66–3.61 (m, 7H)
c	2.85–2.82 (m, 2H)	2.88–2.85 (m, 2H)
d	2.50 (t, 2H)	2.51 (t, 2H)
e	1.94–1.89 (m, 2H)	1.96–1.91 (m, 2H)
f	1.78–1.73 (m, 2H)	1.81–1.76 (m, 2H)
g	1.47 (d, 3H)	1.45 (d, 3H)
h	4.94 (s, 1H)	

**Table 2:** Solubility of PEL and PEL-T at various pH values.

Dissolution Media	PEL (mg/mL)	PEL-T (mg/mL)
pH 1.2	0.03 ± 0.01	654.29 ± 4.53
pH 4.0	0.07 ± 0.04	355.36 ± 5.65
pH 6.8	4.05 ± 0.14	436.67 ± 14.15
water	0.13 ± 0.03	851.60 ± 18.36

Each value is represented as the mean ± S.D. (*n* = 3).

**Table 3:** The permeability coefficients (K_p_) of PEL and PEL-T in the absorptive [A to B] and secretory [B to A] directions, and the efflux ratio across the Caco-2 cell monolayers.

Direction	PEL	PEL-T	*p*-Value
A to B	12.61 ± 1.44	15.35 ± 0.91	0.018
B to A	15.57 ± 4.60	11.65 ± 2.89	0.081
Efflux ratio	1.24	0.76	

Each value is represented as the mean ± S.D. (*n* = 5); K_p_ (cm/h); A, apical; B, basolateral.

## Data Availability

The data presented in this study are available upon request.

## Supplementary Materials

The following are available online at https://www.mdpi.com/article/10.3390/pharmaceutics13050745/s1: Table S1: ^1^H NMR (DMSO-*d_6_* at 400 MHz) chemical shifts (δ) and assignments of the PEL-T; Table S2: ^13^C NMR (DMSO-*d_6_* at 400 MHz) chemical shifts (δ) and assignments of the PEL-T; Table S3: Stress stability (contents); Table S4: Stress stability (degradation products); Figure S1: ^1^H NMR spectra (methanol-*d_4_* at 500 MHz) of (a) PEL and (b) PEL-T; Figure S2: (a) ^1^H NMR spectra (DMSO-*d_6_* at 400 MHz) and (b) ^13^C NMR spectra (DMSO-*d_6_* at 400 MHz) of PEL-T.

## References

[1] NEDRUG Pelubi® (Pelubiprofen) [Korean Prescribing Information]. https://nedrug.mfds.go.kr.

[2] Shin J.S., Baek S.R., Sohn S.I., Cho Y.W., Lee K.-T. (2011). Anti-inflammatory effect of pelubiprofen, 2-[4-(oxocyclohexylidenemethyl)-phenyl]propionic acid, mediated by dual suppression of COX activity and LPS-induced inflammatory gene expression via NF-κB inactivation. J. Cell Biochem..

[3] Song S.H., Chae B.R., Sohn S.I., Yeom D.W., Son H.Y., Kim J.H., Kim S.R., Lee S.G., Choi Y.W. (2016). Formulation of controlled-release pelubiprofen tablet using Kollidon(^®^) SR. Int. J. Pharm..

[4] Asami M., Yamamura M., Nakajima E., Takasaki W., Tanaka Y., Ohtsuki T., Takaichi M., Mitsui T., Yokoshima T. (1995). Metabolic Studies on CS-670, A New 2-Arylpropionic Acid Nonsteroidal Anti-inflammatory Drug (1): Absorption, Metabolism and Excretion of ^14^C-CS-670 in Mice, Rats and Dogs. Drug Metab. Pharmacokinet..

[5] Itoh K., Yamamoto K., Adachi M., Kosaka T., Tanaka Y. (2008). Leukotriene B_4_ 12-hydroxydehydrogenase/15-ketoprostaglandin Delta 13-reductase (LTB_4_ 12-HD/PGR) responsible for the reduction of a double-bond of the alpha,beta-unsaturated ketone of an aryl propionic acid non-steroidal anti-inflammatory agent CS-670. Xenobiotica.

[6] Wolfe M.M., Lichtenstein D.R., Singh G. (1999). BCS toxicity of nonsteroidal antiinflammatory drugs. N. Engl. J. Med..

[7] Schaffer D., Florin T., Eagle C., Marschner I., Singh G., Grobler M., Fenn C., Schou M., Curnow K.M. (2006). Risk of serious NSAID-related gastrointestinal events during long-term exposure: A systematic review. Med. J. Aust..

[8] Goldstein J.L., Correa P., Zhao W.W., Burr A.M., Hubbard R.C., Verburg K.M., Geis G.S. (2001). Reduced incidence of gastroduodenal ulcers with celecoxib, a novel cyclooxygenase-2 inhibitor, compared to naproxen in patients with arthritis. Am. J. Gastroenterol..

[9] Simon L.S., Weaver A.L., Graham D.Y., Kivitz A.J., Lipsky P.E., Hubbard R.C., Isakson P.C., Verburg K.M., Yu S.S., Zhao W.W. (1999). Anti-inflammatory and upper gastrointestinal effects of celecoxib in rheumatoid arthritis: A randomized controlled trial. JAMA.

[10] Emery P., Zeidler H., Kvien T.K., Guslandi M., Naudin R., Stead H., Verburg K.M., Isakson P.C., Hubbard R.C., Geis G.S. (1999). Celecoxib versus diclofenac in long-term management of rheumatoid arthritis: Randomized double-blind comparison. Lancet.

[11] NEDRUG Celebrex® (Celecoxib) [Korean Prescribing Information]. https://nedrug.mfds.go.kr.

[12] Song Y.W., Choi I.A., Baek H.-J., Cho C.S., Lee Y.A., Chung W.T., Park Y.E., Lee Y.J., Park Y.-B., Lee J. (2014). Comparison of the efficacy and safety profiles of a pelubiprofen versus celecoxib in patients with rheumatoid arthritis: A 6-week, multicenter, randomized, double-blind, phase III, non-inferiority clinical trial. BMC Musculoskelet. Disord..

[13] Shin B.J., Kim T.K., Baik J.S., Shim D.M. (2012). Comparison The Safety and The Efficacy between the Group of using Pelubiprofen Tab. and the Group of using Aceclofenac Tab. on Back Pain Patients-Multi Institution, Double Blind, Random Sample. J. Korean Soc. Spine Surg..

[14] Kwak S.S., Lee E.S., Yoon H.Y., Kim C.H., Goo Y.T., Kang M.J., Lee S., Lee B.S., Jeon H.R., Oh C.H. (2018). Immediate release tablet formulation of varenicline salicylate and comparative pharmacokinetic study in human volunteers. Drug Des. Devel. Ther..

[15] Savjani K.T., Gajjar A.K., Savjani J.K. (2012). Drug solubility: Importance and enhancement techniques. ISRN Pharm..

[16] Anderson B.D., Conradi R.A. (1985). Predictive relationships in the water solubility of salts of a nonsteroidal anti-inflammatory drug. J. Pharm. Sci..

[17] Lee T., Wang Y.W. (2009). Initial salt screening procedures for manufacturing ibuprofen. Drug Dev. Ind. Pharm..

[18] Mehlisch D.R., Ardia A., Pallotta T. (2003). Analgesia with ibuprofen arginate versus conventional ibuprofen for patients with dysmenorrhea: A crossover trial. Curr. Ther. Res. Clin. Exp..

[19] Shin D., Lee S.J., Ha Y.-M., Choi Y.-S., Kim J.-W., Park S.-R., Park M.K. (2017). Pharmacokinetic and pharmacodynamic evaluation according to absorption differences in three formulations of ibuprofen. Drug Des. Devel. Ther..

[20] Hubatsch I., Ragnarsson E.G.E., Artursson P. (2007). Determination of drug permeability and prediction of drug absorption in Caco-2 monolayers. Nat. Protoc..

[21] Cho S., Lee J., Yoo Y., Cho M., Sohn S., Lee B.-J. (2021). Improved Manufacturability and In Vivo Comparative Pharmacokinetics of Dapagliflozin Cocrystals in Beagle Dogs and Human Volunteers. Pharmaceutics.

[22] Marvanova P., Padrtova T., Pekarek T., Brus J., Czernek J., Mokry P., Humpa O., Oravec M., Jampilek J. (2016). Synthesis and Characterization of New 3-(4-Arylpiperazin-1-yl)-2-hydroxypropyl 4-Propoxybenzoates and Their Hydrochloride Salts. Molecules.

[23] Bookwala M., Thipsay P., Ross S., Zhang F., Bandari S., Repka M.A. (2018). Preparation of a crystalline salt of indomethacin and tromethamine by hot melt extrusion technology. Eur. J. Pharm. Biopharm..

[24] Vadivelu N., Gowda A.M., Urman R.D., Jolly S., Kodumudi V., Maria M., Taylor R., Pergolizzi J.V. (2015). Ketorolac tromethamine—Routes and clinical implications. Pain Pract..

[25] Wang T., Wu G., Wang J., Cui Y., Ma J., Zhu Z., Qiu J., Wu J. (2020). Comparison of single-dose fosfomycin tromethamine and other antibiotics for lower uncomplicated urinary tract infection in women and asymptomatic bacteriuria in pregnant women: A systematic review and meta-analysis. Int. J. Antimicrob. Agents.

[26] Crowe A., Wright C. (2012). The impact of P-glycoprotein mediated efflux on absorption of 11 sedating and less-sedating antihistamines using Caco-2 monolayers. Xenobiotica.

[27] Nahas G.G., Sutin K.M., Fermon C., Streat S., Wiklund L., Wahlander S., Yellin P., Brasch H., Kanchuger M., Capan L. (1998). Guidelines for the treatment of acidaemia with THAM. Drugs.

[28] Mauleón D., Artigas R., García M.L., Carganico G. (1996). Preclinical and clinical development of dexketoprofen. Drugs.

[29] Song W.H., Yeom D.W., Lee D.H., Lee K.M., Yoo H.J., Chae B.R., Song S.H., Choi Y.W. (2014). In situ intestinal permeability and in vivo oral bioavailability of celecoxib in supersaturating self-emulsifying drug delivery system. Arch. Pharm. Res..

[30] Liu L., Zou D., Zhang Y., Zhang Q., Feng Y., Guo Y., Liu Y., Zhang X., Cheng G., Wang C. (2020). Pharmaceutical salts/cocrystals of enoxacin with dicarboxylic acids: Enhancing in vitro antibacterial activity of enoxacin by improving the solubility and permeability. Eur. J. Pharm. Biopharm..

[31] Ling H., Luoma J.T., Hilleman D. (2013). A Review of Currently Available Fenofibrate and Fenofibric Acid Formulations. Cardiol. Res..

[32] Charalabidis A., Sfouni M., Bergström C., Macheras P. (2019). The Biopharmaceutics Classification System (BCS) and the Biopharmaceutics Drug Disposition Classification System (BDDCS): Beyond guidelines. Int. J. Pharm..

[33] Vogt M., Kunath K., Dressman J.B. (2008). Dissolution enhancement of fenofibrate by micronization, cogrinding and spray-drying: Comparison with commercial preparations. Eur. J. Pharm. Biopharm..

[34] Bosselmann S., Williams R.O. (2012). Has nanotechnology led to improved therapeutic outcomes?. Drug Dev. Ind. Pharm..

[35] (2011). Trilipix® (fenofibric acid delayed release capsules). Full Prescribing Information.

