# Supplementary material for: Development of Pelubiprofen Tromethamine with Improved Gastrointestinal Safety and Absorption
Source: Pharmaceutics. 2021 May 18;13(5):745. doi: 10.3390/pharmaceutics13050745 (PMC8158122; doi:10.3390/pharmaceutics13050745)

pharmaceutics

MDPI

Supplementary Materials: Development of Novel Pelubiprofen Tromethamine Salt with Improved Gastrointestinal Safety and Absorption to the Commercial Pelubiprofen

Ji Yeon Park, Dong Ho Oh, Sang-Wook Park, Bo Ram Chae, Chul Woo Kim, Sang Heon Han, Heon Jong Sin, Soo Bin Yeom, Da Yeong Lee, Min Kyu Park, Jun-Bom Park, Kyung-Tae Lee

Table S1. ^{1}H NMR (DMSO-d_6 at 400 MHz) chemical shifts (δ) and assignments of the PEL-T.

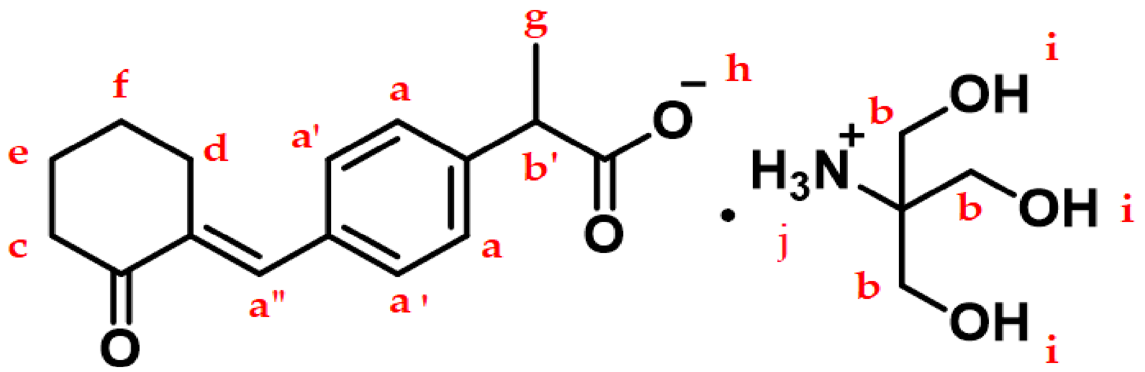

Assignments	Chemical Shifts	No. of Hydrogen
a, a′, a″	7.30–7.34	5
i, j	5.33	6
b′	3.44–3.46	1
b	3.37	6
c	2.77	2
d	2.40–2.48	2
e	1.82	2
f	1.67	2
g	1.28–1.30	3

Table S2. ^{13}C NMR (DMSO-d_6 at 400 MHz) chemical shifts (δ) and assignments of the PEL-T.

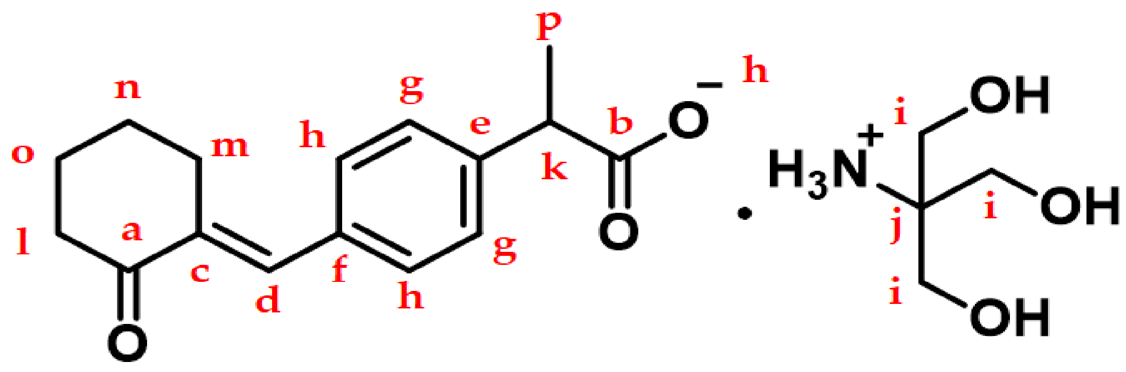

Assignments	Chemical shifts	No. of carbons
a	200.62	1
b	177.62	1
c	145.39	1
d	136.39	1
e	134.81	1
f	133.15	1
g	130.52	2
h	128.13	2
i	61.00	3
j	60.21	1
k	47.73	1
l	40.42	1
m	28.89	1
n	23.71	1
o	23.13	1
p	19.83	1

Table S3. Stress stability (contents).

Storage Conditions	Time (days)	Contents (%)[a]	
		PEL	PEL-T
80 ± 5 °C	0	98.6 ± 0.8	98.4 ± 0.1
	14	98.2 ± 0.6	98.1 ± 0.1
	21	97.1 ± 0.9	98.2 ± 0.2
RH 90 ± 5%	0	98.6 ± 0.8	98.4 ± 0.1
	14	98.9 ± 0.4	98.6 ± 0.2
	21	98.5 ± 0.4	98.3 ± 0.2

Each value represents the mean ± S.D. ($n = 3$).; [a] Acceptance criteria was set for PEL contents of 95%–105%.; PEL, pelubiprofen; PEL-T, pelubiprofen tromethamine; RH, relative humidity

Table S4. Stress stability (degradation products).

Storage conditions	Time (days)	Degradation Products (%)[a]			
		Impurity A		Total peaks	
		PEL	PEL-T	PEL	PEL-T
80 ± 5 °C	0	0.19 ± 0.03	0.15 ± 0.02	0.24 ± 0.03	0.24 ± 0.03
	14	0.22 ± 0.03	0.16 ± 0.02	0.31 ± 0.03	0.24 ± 0.01
	21	0.21 ± 0.02	0.13 ± 0.03	0.87 ± 0.05	0.35 ± 0.04
RH 90 ± 5%	0	0.19 ± 0.03	0.15 ± 0.02	0.24 ± 0.03	0.24 ± 0.03
	14	0.20 ± 0.03	0.15 ± 0.03	0.26 ± 0.01	0.24 ± 0.01
	21	0.16 ± 0.02	0.12 ± 0.02	0.33 ± 0.02	0.45 ± 0.06

Each value represents the mean ± S.D. ($n = 3$). [a]Acceptance criteria was set for degradation products of less than 0.5% for the impurity A and 1.0% for the total peaks in the relative peak area.

Figure S1. ^{1}H NMR spectra (methanol-d_4 at 500 MHz) of (**a**) PEL and (**b**) PEL-T.

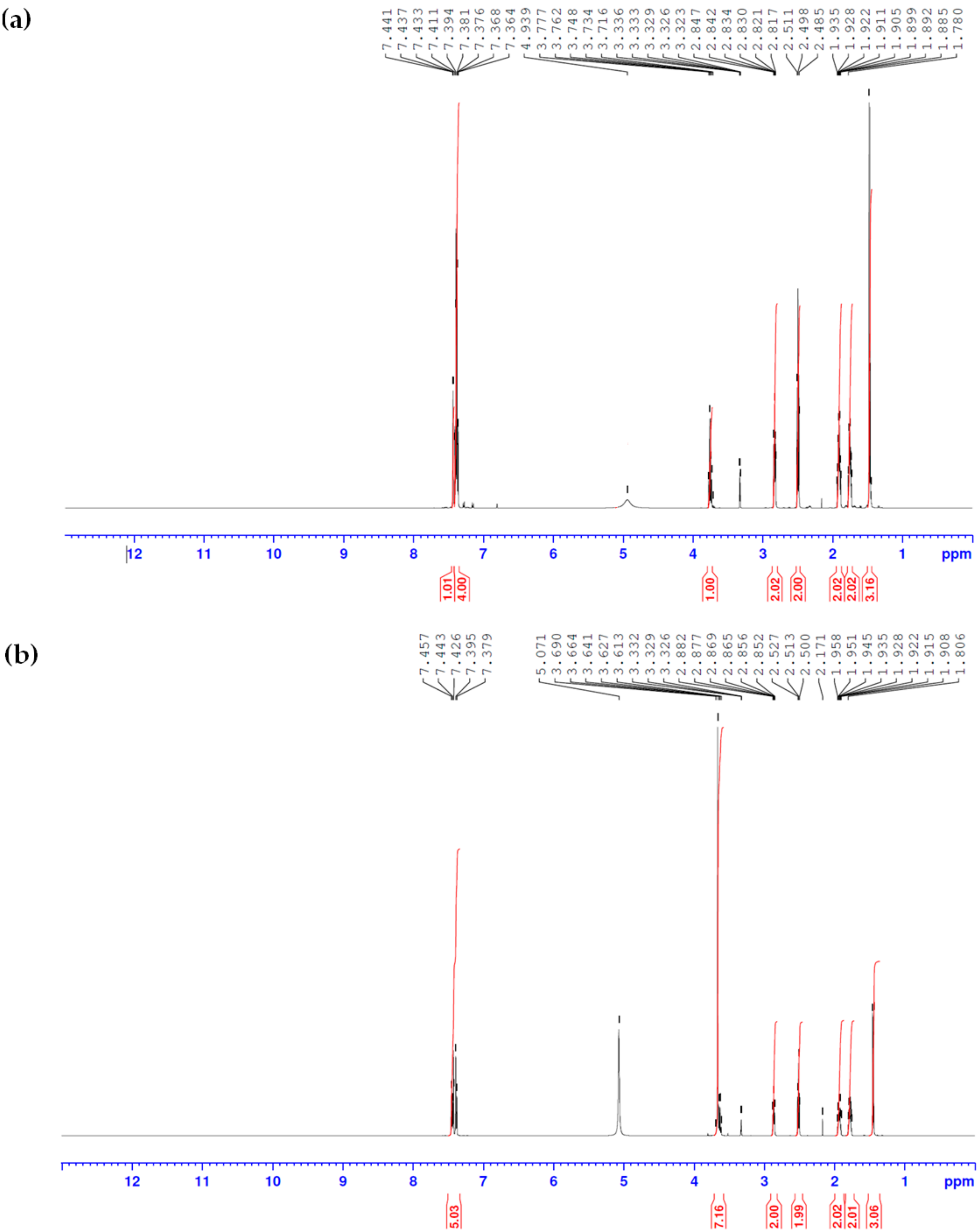

Figure S2. (**a**) ^{1}H NMR spectra (DMSO-d_6 at 400 MHz) and (**b**) ^{13}C NMR spectra (DMSO-d_6 at 400 MHz) of PEL-T.

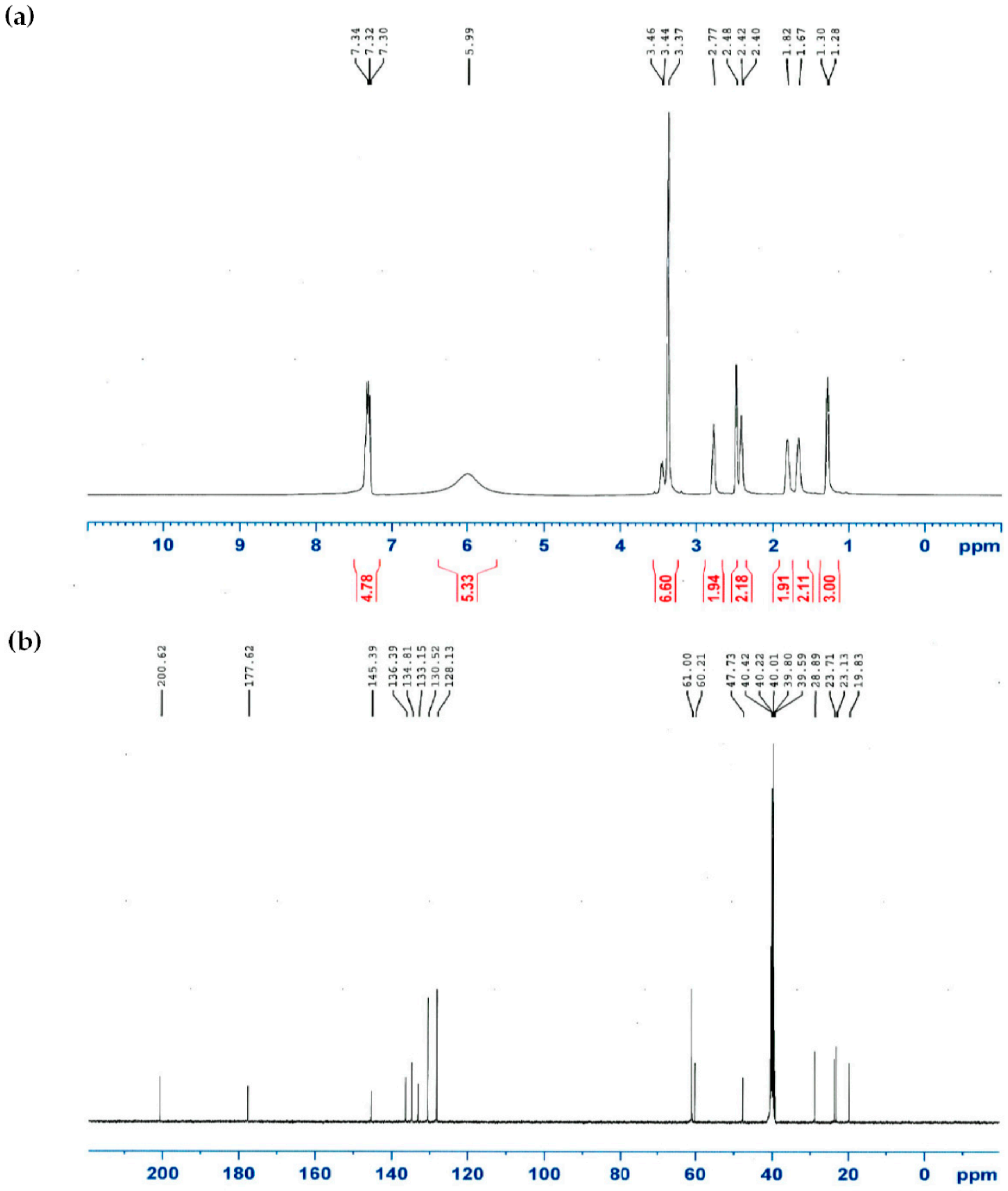

Supplement: Supplementary file 1 [file pharmaceutics-13-00745-s001.zip › pharmaceutics-1220198-supplementary.pdf]
